# Quick and easy sample preparation without resin embedding for the bone quality assessment of fresh calcified bone using fourier transform infrared imaging

**DOI:** 10.1371/journal.pone.0189650

**Published:** 2018-02-06

**Authors:** Hiromi Kimura-Suda, Masahiko Takahata, Teppei Ito, Tomohiro Shimizu, Kyosuke Kanazawa, Masahiro Ota, Norimasa Iwasaki

**Affiliations:** 1 Graduate School of Photonics Science, Chitose Institute of Science and Technology, Chitose, Hokkaido, Japan; 2 Department of Orthopedic Surgery, School of Medicine, Hokkaido University, Sapporo, Japan; Indiana University Purdue University at Indianapolis, UNITED STATES

## Abstract

Fourier transform infrared (FTIR) imaging is a powerful tool for the assessment of bone quality; however, it requires the preparation of thin bone sections. Conventional poly(methyl methacrylate) (PMMA) embedding for the preparation of sections takes more than two weeks and causes denaturation of the bone. Development of a quick and easy sample preparation technique without denaturation is needed for accurate clinical evaluation of fresh calcified bone using FTIR imaging. Frozen sectioning allows the quick and easy preparation of thin sections without denaturation, but it requires a substrate with good chemical resistance and improved heat shock resistance. Polypropylene (PP) film afforded both good chemical resistance and greater heat shock resistance, and the 4-μm-thick PP film coated with glue was thin enough for the IR beam to pass through it, while the optical anisotropy of infrared bands overlapping with PO_4_^3-^ band was negligible. The bone quality of femoral thin sections prepared by the conventional PMMA embedding and sectioning procedure (RESIN-S) or the newly developed frozen sectioning procedure (FROZEN-S) was evaluated by FTIR imaging. The mineral-to-matrix ratio and crystallinity in the RESIN-S sections were higher than those in the FROZEN-S sections, whereas the carbonate-to-phosphate ratio in the RESIN-S sections was lower than that in the FROZEN-S sections. In RESIN-S, the increased mineral-to-matrix ratio could be caused by dehydration, and the increased crystallinity and decreased carbonate-to-phosphate ratio might be consequence of dissolution of bone mineral during PMMA embedding. Therefore, the combined use of PP film coated with glue and the frozen sectioning procedure without denaturation appears well suited to the assessment of the bone quality of fresh calcified bone using FTIR imaging.

## Introduction

Osteoporosis is a bone disease characterized by increased skeletal fragility due to a reduction in bone strength. Bone strength is derived from a combination of bone mass (bone mineral content or bone mineral density: BMD) and bone quality, which results from a combination of material and structural properties [1]. Bone is basically composed of 65% minerals (apatite containing approximately 7% carbonate), 20–25% type I collagen, 10% water and a small amount of non-collagenous organic material [1, 2]. The following factors are generally accepted to be related to bone quality: rate of turnover, architecture/geometry of the trabecular and cortical bone, properties of the mineral/collagen matrix and accumulation of microdamage [1].

Fourier transform infrared (FTIR) spectroscopic imaging is a powerful tool for assessing bone quality, including parameters such as the mineral-to-matrix ratio (degree of mineralization), carbonate-to-phosphate ratio (degree of carbonation), crystallinity (size/strain perfection) [3, 4], mineral maturity (transformation of nonapatitic into apatitic domains) [3], collagen cross-links, collagen fiber orientation, and hydroxyapatite crystal orientation [3, 5–11]. FTIR images are collected in one of following modes: transmission mode, reflection mode, or attenuated total reflection (ATR) mode. Generally, the transmission mode is used to assess bone quality because of its high quantitativity [12]. The assessment of bone quality using FTIR imaging in the transmission mode requires the preparation of sufficiently thin bone sections of less than 5 μm as the wavenumber range from 1800 to 800 cm^-1^ in the FTIR spectrum is usually used to evaluate bone quality [9, 12–14]. In the conventional method for preparing thin bone sections using the poly(methyl methacrylate) (PMMA) embedding procedure, fresh bone has to be fixed in 70% ethanol (EtOH) for 1–2 days, dehydrated by soaking for 48 hrs with several repeated changes of 100% EtOH, and substituted in methyl methacrylate (MMA) for 48 hrs, followed by polymerization for over a week to obtain a PMMA block [15]. Thus, the PMMA embedding procedure for bone takes more than two weeks, which hampers the assessment of bone quality using FTIR imaging as a clinical measurement. In addition, that process exposes the bone to high concentration of EtOH and MMA, causing denaturation of collagen and other proteins [4, 15–19]. Previously, the effects of fixation and embedding on bone were determined by FTIR and Raman spectroscopies, and slight changes in the shapes of both the phosphate (PO_4_^3-^) band derived from hydroxyapatite and the amide I band derived from collagen were observed in both the FTIR and Raman spectra after exposure to the solvent and embedding medium [4, 15, 18, 20]. Pleshko et al. demonstrated that EtOH fixation altered the secondary structure of the protein in 5-week-old rat femurs, and formalin fixation altered it in 20-day-old fetal rat femurs; however, the crystallinity in the 5-week-old rat femurs was not altered by EtOH fixation [18]. Moreover, Aparico et al. reported that PMMA penetrated into lowly mineralized tissues, and the PMMA spectrum overlapped with the mineral and matrix spectra, making the subtraction of the spectra more difficult [15]. Yeni et al. reported that the degrees of mineralization, carbonation, and crystallinity were affected by EtOH fixation and the embedding medium. Therefore, the assessment of bone quality using FTIR imaging requires the use of a frozen sectioning procedure that does not require fixation in order to achieve more accurate measurements. Such a frozen sectioning procedure without fixation maybe shorten the time and cost required in comparison to the PMMA embedding and sectioning procedure.

FTIR imaging for the assessment of bone quality involves the mounting of thin bone sections on a BaF_2_ window, which has very low solubility and a good spectral range, but is fragile, susceptible to thermal shock and expensive. It is highly likely that the BaF_2_ substrate will break at the low temperatures (-30˚C) necessary for mounting frozen sections. Therefore, the BaF_2_ substrate is not particularly suited to a conventional frozen section procedure, which needs the substrate to be placed directly in a freezer. Low-cost substrates, such as regular glass slides cannot be used in the transmission mode [21] as the IR beam cannot pass through glass in the range from 1800 to 800 cm^-1^. Therefore, we used 4-μm-thick polypropylene (PP) film as a substrate for the frozen sections. PP possesses a number of advantages in that it shows good chemical resistance and better heat shock resistance, and is translucent, tough, and inexpensive. PP has a wide range of applications in films and packaging for foods, tubes and bottles for solutions, fibers, biomaterials, and disposable laboratory supplies. Moreover, the transparent, high-purity PP thin film is used as a sample-holding film for elemental analysis by x-ray fluorescence (XRF) [22]. On the other hand, it is possible that the orientation of the polymer main-chains in the PP thin film results in optical anisotropy. In FTIR spectroscopy, a polarizer is sometimes used to obtain information on molecular orientation. We previously determined the orientation of the collagen fibers in rat femur sections on the BaF_2_ substrate using FTIR imaging with a polarizer [7]. We found that the optical anisotoropy of the PP film has the potential to affect the data with regards to molecular orientation in bone mounted on the PP film during FTIR imaging with a polarizer.

In this study, we characterized the PP film coated with glue (PP film + glue) and fresh bone mounted on the PP film using FTIR imaging with a polarizer and aimed to demonstrate that the combined use of the thin PP film coated with glue as a substrate and the frozen sectioning procedure without denaturation of proteins by solvent or resin was particularly suited to the assessment of the bone quality of fresh calcified bone using FTIR imaging in transmission mode, and that the newly developed sample preparation procedure afforded a powerful technique for clinical evaluation due to reductions in time and cost.

## Materials and methods

### PP film coated with glue

**Fig 1:**
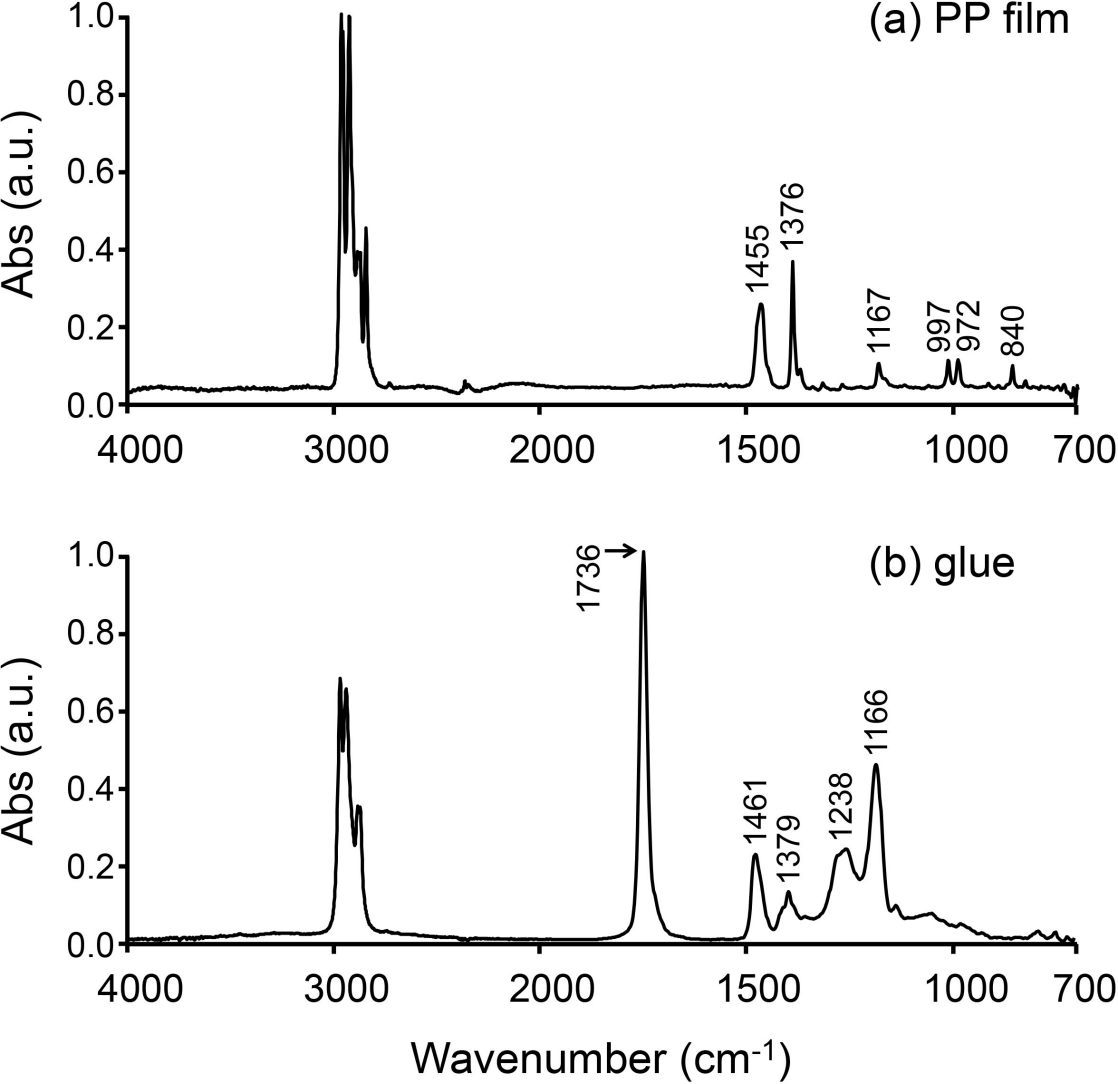
FTIR spectra of PP film and glue. (a) FTIR spectrum of the PP film. (b) FTIR spectrum of the glue. The spectra were extracted from the FTIR maps and normalized against 1 absorbance with respect to the highest band.

Four-μm-thick PP film (Ultralene, SPEX SamplePrep, NJ, USA) was used as a substrate for mounting thin bone sections prepared by a frozen sectioning procedure (Fig 1). The film was coated with commercial glue (Cryoglue type I, Leica Microsystems, Tokyo, Japan), which was used to fix the thin bone sections (Fig 1). The glue was diluted with n-hexane (glue:n-hexane = 9:1), and spread using a spin coater (MS-A100, Mikasa, Tokyo, Japan) at 2000 rpm for 10 sec at room temperature. All PP film, glue, and PP film + glue samples were mounted on BaF_2_ substrates, and their FTIR spectra were collected using a Spotlight 400 FTIR Imaging System (PerkinElmer, MA, USA) with a single element Mercury Cadmium Telluride (MCT) detector in transmission mode at a spectral resolution of 4 cm^-1^, aperture size of 100 μm x 100 μm, and with 32 scans in the mid-IR region from 4000 to 680 cm^-1^. The background spectra were collected through the BaF_2_ window. FTIR spectra underwent baseline correction, which was automatically applied across the whole spectrum (4000 to 680 cm^-1^), using Spectrum 10 software (PerkinElmer, MA, USA). The spectra were normalized against 1 absorbance with respect to the highest band.

### Angle-dependent FTIR spectrum of PP film

A 100 μm x 100 μm area of the PP film on the BaF_2_ window was determined using the FTIR imaging system with a single element MCT detector and a wire grid polarizer (ST Japan Inc., Tokyo, Japan) in order to characterize the angle dependence of the infrared (IR) absorption. The analytical conditions for the FTIR instrument were as described above. The polarizer was rotated in the range from 0 to 180° in increments of 10°.

### Bone

Six 12-week-old BALB/cA mice (CLEA Japan, Inc., Tokyo, Japan) were euthanized by cervical disolocation with an intraperitoneal injection of 5 μL/g cocktail of ketamine (20 mg/mL) and xylazine (2 mg/mL). After euthanasia, femurs were obtained by dislocating hip and knee joints, and trimmed to remove soft tissue. Next, they were immediately washed with physiological saline solution to remove blood. Thin sections of three femurs were prepared using EtOH fixation followed by the PMMA embedding procedure (RESIN-S). Additional thin sections of the other three femurs were prepared using a frozen sectioning procedure (FROZEN-S) without EtOH fixation.

The Ethics Review Committee for Animal Experimentation of Hokkaido University approved the experimental protocol of this study (15–0077).

### PMMA embedding and sectioning procedure

Three femurs were fixed in 70% EtOH and trimmed to remove soft tissues (mainly muscle). They were then dehydrated in ascending grades of EtOH, defatted in an acetone/methyl methacrylate monomer mixture (1:2), and embedded in MMA (Wako Chemicals, Kanagawa, Japan) without decalcification. Longitudinal frontal plane sections (3-μm-thick) of the distal femur (RESIN-S) were cut using a microtome (Rotary microtome, RM2255, Leica, Germany). Thereafter, they were sandwiched between two glass plates and compressed to provide a flat surface in order to obtain a good FTIR spectrum. The sections were mounted on a BaF_2_ window (Pier Optics, Japan) to assess bone quality by FTIR imaging in transmission mode.

### Frozen sectioning procedure

**Fig 2:**
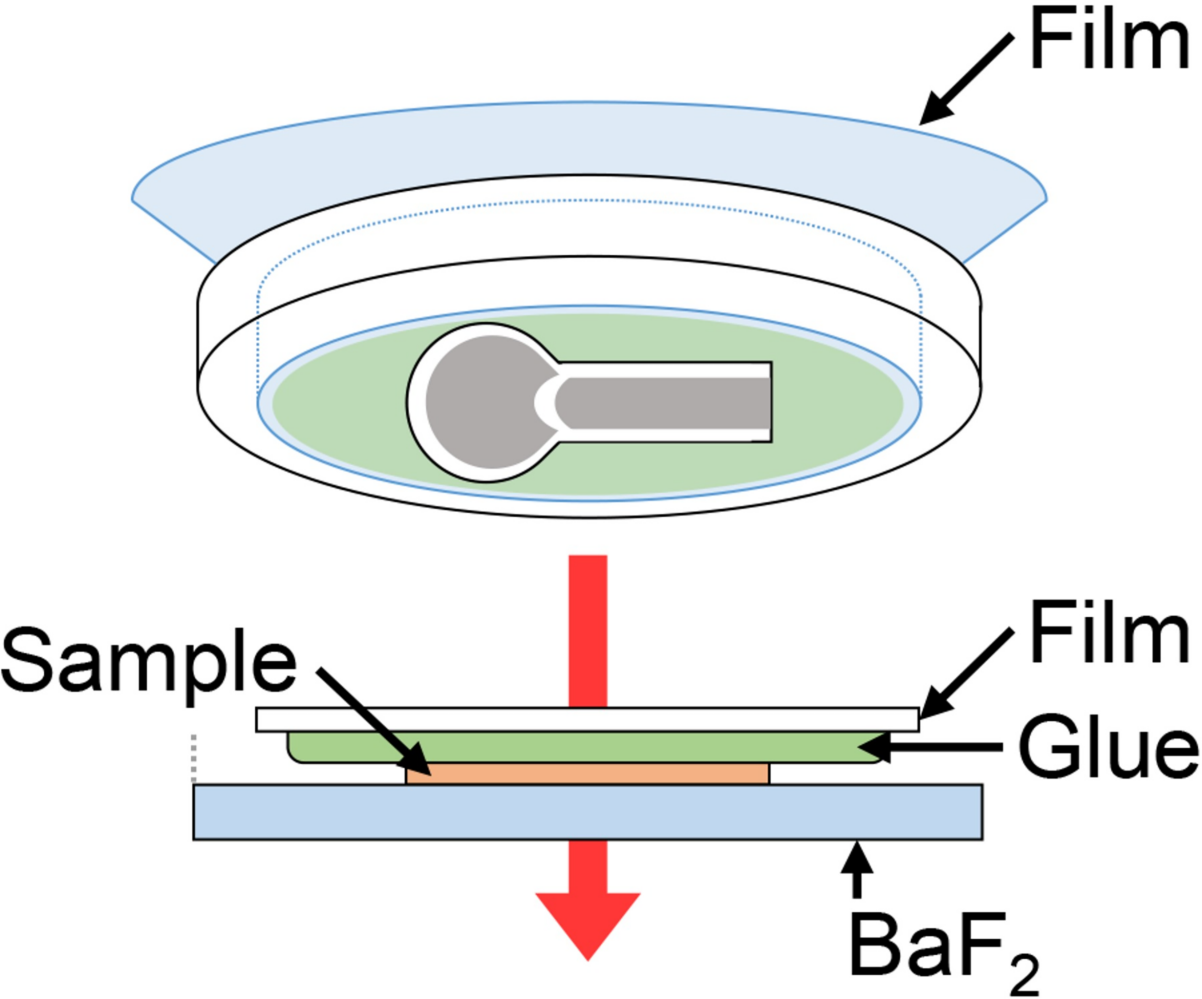
Schematic overview of femur section mounting. The frozen section on the glue-coated side of the PP film was placed face down on the regular BaF_2_ substrate in order to fix the sample position, while leaving a space in which the BaF_2_ substrate not covered with the PP film + glue.

Three femurs were trimmed to remove soft tissues. They were immediately placed in Super Cryo Embedding Medium (SCEM, Leica Microsystems; Tokyo) in stainless steel cups and frozen by immersion in an n-hexane (Wako Pure Chemical Industries, Osaka, Japan) and dry ice slurry at room temperature. The frozen tissue block was attached to a cryostat microtome (CM 3050S, Leica, Solms, Germany) at -30°C and sliced to prepare longitudinal frontal plane sections (3-μm-thick) using a tungsten carbide knife. The sliced thin sections in the cryostat (FROZEN-S) were mounted on the glue-coated surface of the PP film and rinsed with distilled water to remove extra SCEM and dried at room temperature (S1 Fig). We used two hoops to keep the film taut in order to mount the froze sections without deformation. The section on the glue-coated side of the PP film was face down on the regular BaF_2_ substrate, which was used simply as a sample stage to fix the sample position (Fig 2).

### Bone quality assessment by FTIR imaging

FTIR images of the RESIN-S and FROZEN-S, which were mounted on the BaF_2_ substrate, were collected using the FTIR imaging system with a linear array MCT detector in transmission mode at a spectral resolution of 4 cm^-1^, 2 scans/pixel, and pixel size of 25 μm x 25 μm in the region from 4000 to 680 cm^-1^. The background spectra were recorded on the BaF_2_ window. Spectrum IMAGE (PerkinElmer, MA, USA) was used to display the set of FTIR spectra as images in which areas of high absorbance for the PO_4_^3-^ band are shown as white (or red) and areas of lower absorbance for the PO_4_^3-^ band are shown as dark blue. The chemical distribution images of the components in the sample were generated using characteristic FTIR bands of the functional groups. We used the PO_4_^3-^ band (1142–916 cm^-1^) to show the FTIR image of the femur. Thirty FTIR spectra were extracted from each FTIR image of the femur for the cortical and trabecular bone separately, underwent baseline correction (4000–680 cm^-1^), PMMA or PP film + glue subtraction, and normalized against 1 absorbance. The following bone quality parameters were then calculated from the FTIR spectra using Spectrum 10 software. The common parameters of bone quality associated with the FTIR spectral ranges are summarized in Table 1 [9, 23]. The mineral-to-matrix ratio (PO_4_^3-^/amide I) was calculated by integrating the area of the PO_4_^3-^ band (1142–916 cm^-1^) and dividing it by the area of the amide I band (1719–1592 cm^-1^). The carbonate-to-phosphate ratio (CO_3_^2-^/PO_4_^3-^) was calculated by integrating the area of the CO_3_^2-^ band (893–855 cm^-1^) and dividing it by the area of the PO_4_^3-^ band. The crystallinity (1030 cm^-1^/1020 cm^-1^) was calculated by dividing the height of the PO_4_^3-^ band (baseline:1142–916 cm^-1^) at 1030 cm^-1^ by the height of the PO_4_^3-^ band at 1020 cm^-1^. Bone quality values were averaged for 30 FTIR spectra extracted from each FTIR image for cortical and trabecular bone separately, and then averaged for n = 3 per group for cortical and trabecular bone. Statistical analysis was performed by unpaired t-test (n = 3) to compare RESIN-S and FROZEN-S, and the *p* values are shown above the plots.

**Table 1:** Bone chemical composition parameters and wavenumbers of FTIR bands.

Chemical composition parameters	RESIN-S (cm^-1^)	FROZEN-S (cm^-1^)
Carbonate (CO_3_^2-^)	893–855	893–855
Phosphate (PO_4_^3-^)	1142–916	1142–916
Matrix (amide I)	1719–1592	1719–1592
Mineral/Matrix (PO_4_^3-^ / amide I)	1142–916 / 1719–1592	1142–916 / 1719–1592
Carbonate/Phosphate (CO_3_^2-^ / PO_4_^3-^)	893–855 /1142-916	893-855/ 1142–916
Crystallinity	1030 / 1020	1030 / 1020

## Results

### PP film coated with glue

**Fig 3:**
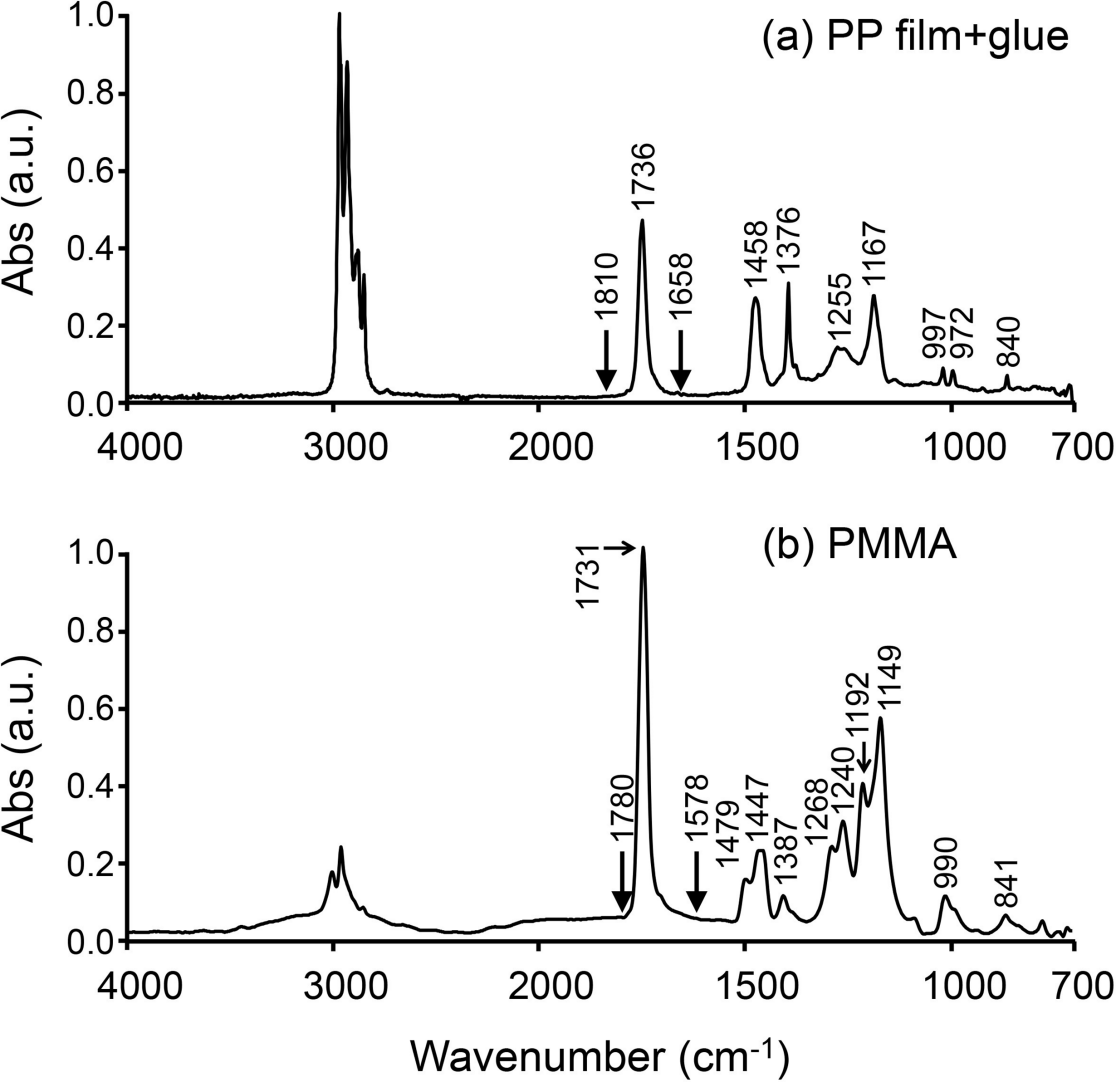
FTIR spectra of PP film + glue and PMMA. These spectra were extracted from the FTIR maps and normalized against 1 absorbance with respect to the highest band.

The positions of the absorption bands for the PP film + glue and PMMA were determined by FTIR imaging system (Fig 3). In the FTIR spectrum of the PP film + glue (a), both intense absorption bands at 1736 cm^-1^ (C = O) and 3051–2752 cm^-1^ (CH_2_, CH_3_) and medium/weak absorption bands (1458, 1376, 1255, 1167, 997, 972, 840 cm^-1^) were observed. The most intense bands (3051–2752 cm^-1^) was not saturated. These results indicated that the 4-μm-thick PP film was thin enough for the IR beam to pass through it. The region of the C = O band in the FTIR spectrum of PP film + glue was from 1810 to 1658 cm^-1^, and that for PMMA was from 1780 to 1578 cm^-1^. The C = O band for PMMA overlapped with the amide I band (1719–1592 cm^-1^) for the bone more than that for the PP film + glue. On the other hand, the absorption bands at 1458, 1376, 1255, 1167, and 840 cm^-1^ in the FTIR spectrum of the PP film + glue did not overlap with either the amide I or PO_4_^3-^ bands (1142–916 cm^-1^), which were bone chemical composition parameters for the assessment of bone quality (Table 1). The absorption bands at 997 and 972 cm^-1^ were extremely weak and negligible; however, those bands were found to overlap with PO_4_^3-^.

### Angle-dependent FTIR spectrum of PP film

**Fig 4:**
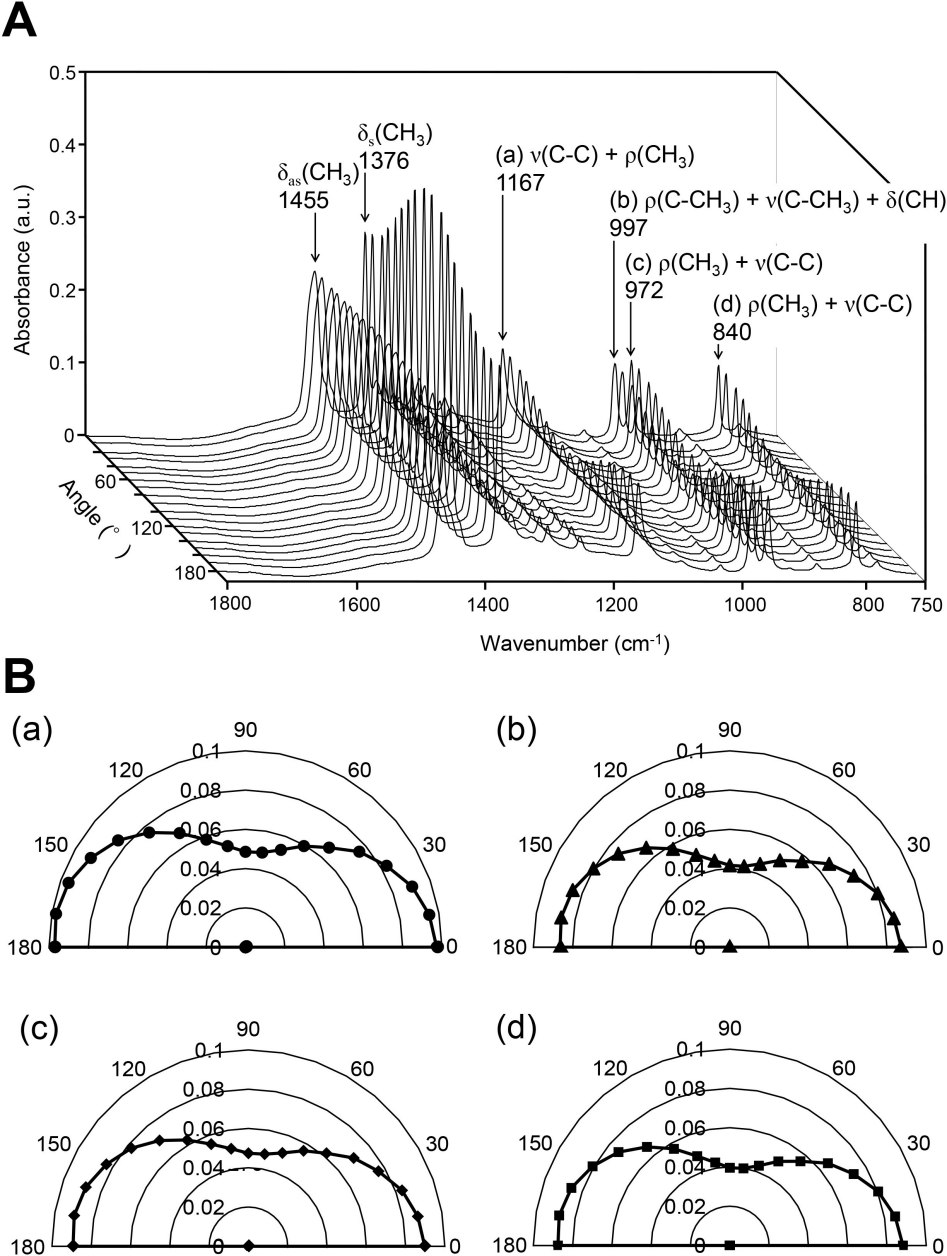
A series of FTIR polarization spectra and their angle-dependent absorbance. (A) The FTIR polarization spectra were collected in the range from 0 to 180° in increments of 10°. (B) The angle-dependent absorbance was obtained at 1167 cm^-1^, 997 cm^-1^, 972 cm^-1^, and 840 cm^-1^. (a) Absorption at 1167 cm^-1^ is a combination of the C-C stretching [ν(C-C)] and CH_3_ rocking vibration [ρ(CH_3_)]. (b) Absorption at 997 cm^-1^ is a combination of the C-CH_3_ rocking vibration [ρ(C-CH_3_)], C-CH_3_ stretching [ν(C-CH_3_)] and CH deformation band [δ(CH)]. Absorption at both 972 cm^-1^ and 840 cm^-1^ are combinations of the CH_3_ rocking vibration [ρ(CH_3_)] and C-C stretching [ν(C-C)].

A series of FTIR spectra of the PP film were collected using a polarized IR beam to evaluate the optical anisotropy (Fig 4A). The absorbance of the CH_3_ band at 1376 cm^-1^ was dependent on the polarizing angle and was highest at around 90°. The absorbance of the bands at 1167 cm^-1^ (a), 997 cm^-1^ (b), 972 cm^-1^ (c), and 840 cm^-1^ (d) was also dependent on the polarizing angle; however, their absorbance was the lowest at around 90°. The lowest absorbance of (a), (b), (c), and (d) was approximately 45–50% of their highest absorbance (Fig 4B).

### Effects of the sectioning procedures on bone quality

**Fig 5:**
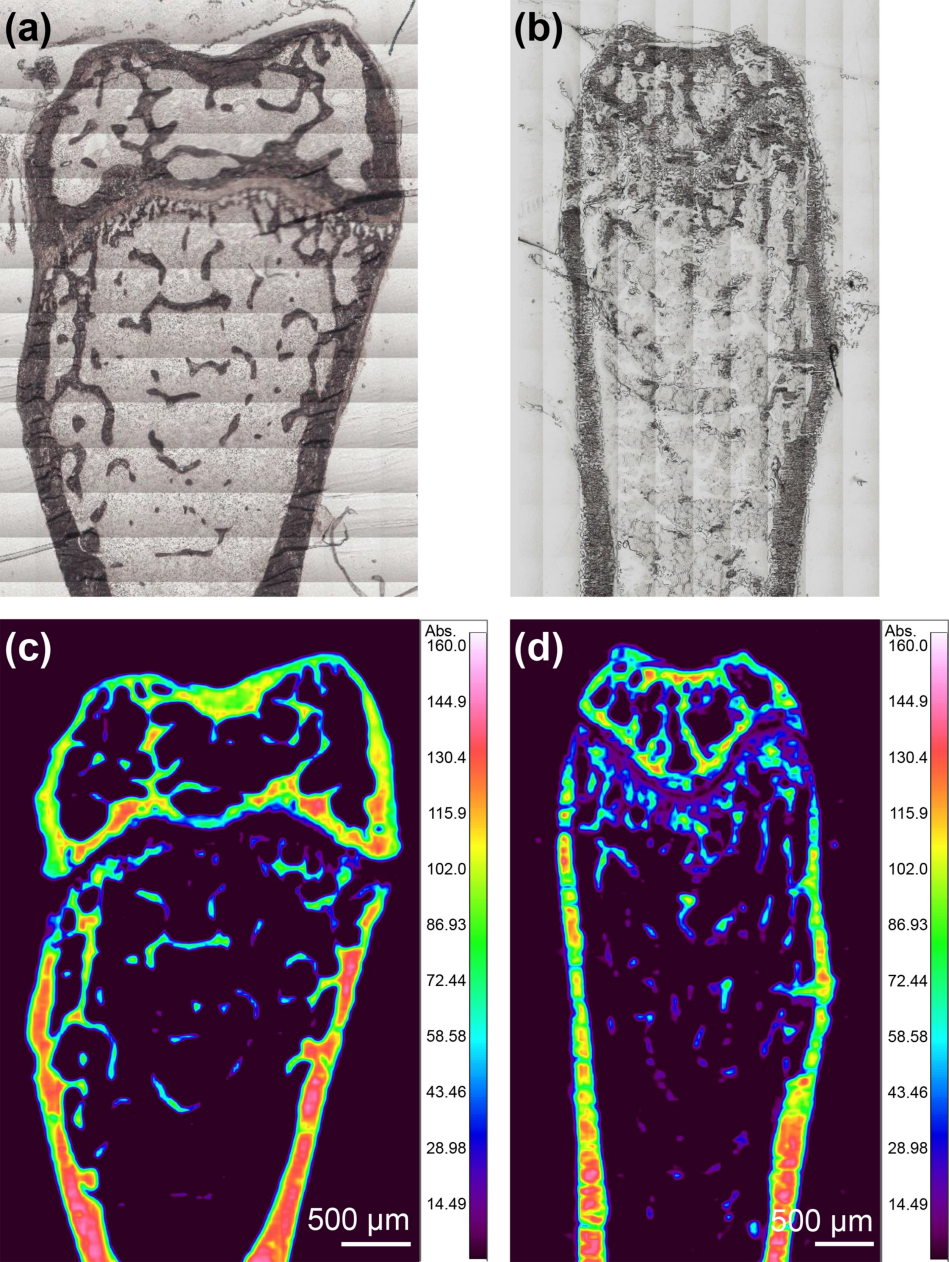
Visible and FTIR images of mouse femurs. (a) Visible image of RESIN-S. (b) Visible image of FROZEN-S. (c) FTIR image of the distribution of the PO_4_^3-^ band in RESIN-S. (d) FTIR image of the distribution of the PO_4_^3-^ band in FROZEN-S.

Longitudinal frontal plane sections of the mouse femurs were prepared using two different procedures (RESIN-S, FROZEN-S) and FTIR imaging was used to examine the effects of the embedding procedure on bone quality. Visible images of the RESIN-S (Fig 5A) and FROZEN-S (Fig 5B) sections showed that those sections were not deformed by sectioning and mounting on the BaF_2_ window, with the cortical and trabecular bone clearly observable. FTIR images of the distribution of the PO_4_^3-^ band in the RESIN-S (Fig 5C) and FROZEN-S (Fig 5D) sections indicated that the distribution of the hydroxyapatite was heterogeneous. To compare the FROZEN-S and RESIN-S sections, the mineral-to-matrix ratio, carbonate-to-phosphate ratio, and crystallinity were calculated using the FTIR spectra extracted from the FTIR images. The FTIR spectra of the femur sections on the PP film + glue were not saturated in the region from 2000 cm^-1^ to 700 cm^-1^: however, the total thickness of the samples (femur sections + PP film + glue) was thick (~7 μm) (S2 Fig).

**Fig 6:**
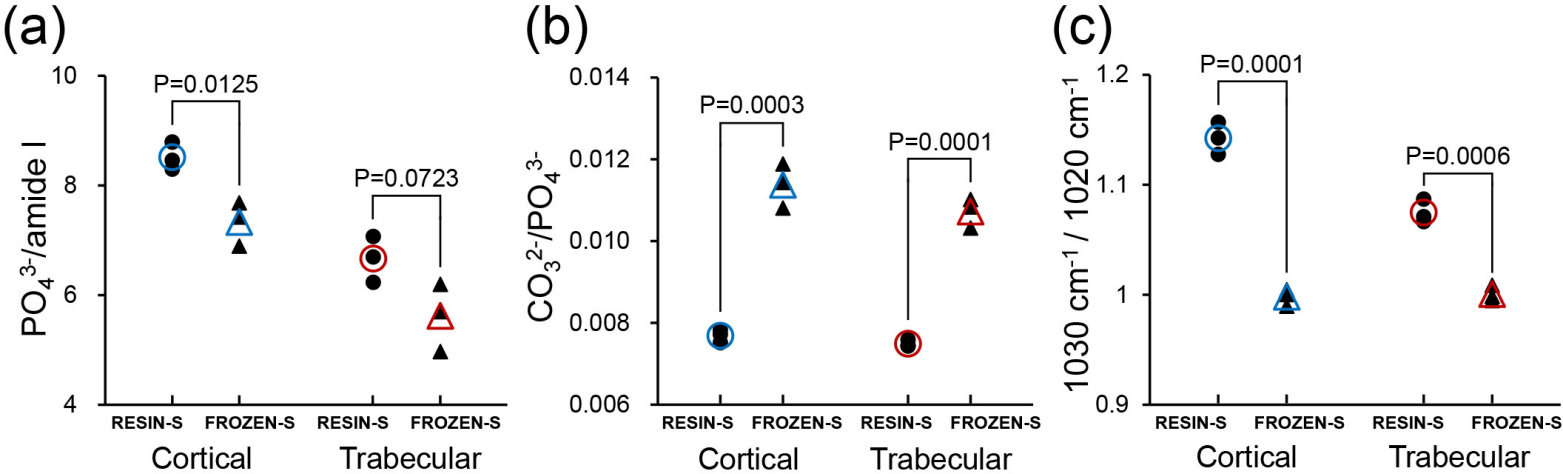
Bone quality of femur sections prepared by different sectioning procedures. (a) Mineral-to-matrix ratio in RESIN-S and FROZEN-S. (b) Carbonate-to-phosphate ratio in RESIN-S and FROZEN-S. (c) Crystallinity in RESIN-S and FROZEN-S. Small closed circles and triangles represent the average bone quality values for 30 FTIR spectra extracted from each femur for the cortical and trabecular bone, respectively. Blue open circles and triangles represent the average of 3 plots for cortical bone. Red open circles and triangles represent the average of 3 plots for trabecular bone. Statistical analysis was performed by unpaired t-test (n = 3) to compare RESIN-S and FROZEN-S, and the *p* values are shown above the plots.

As shown in Fig 6, both the mineral-to-matrix ratio (a) and crystallinity (c) in the RESIN-S sections were higher than those in the FROZEN-S sections, whereas the carbonate-to-phosphate ratio (b) in the RESIN-S sections was lower than that in the FROZEN-S sections.

**Fig 7:**
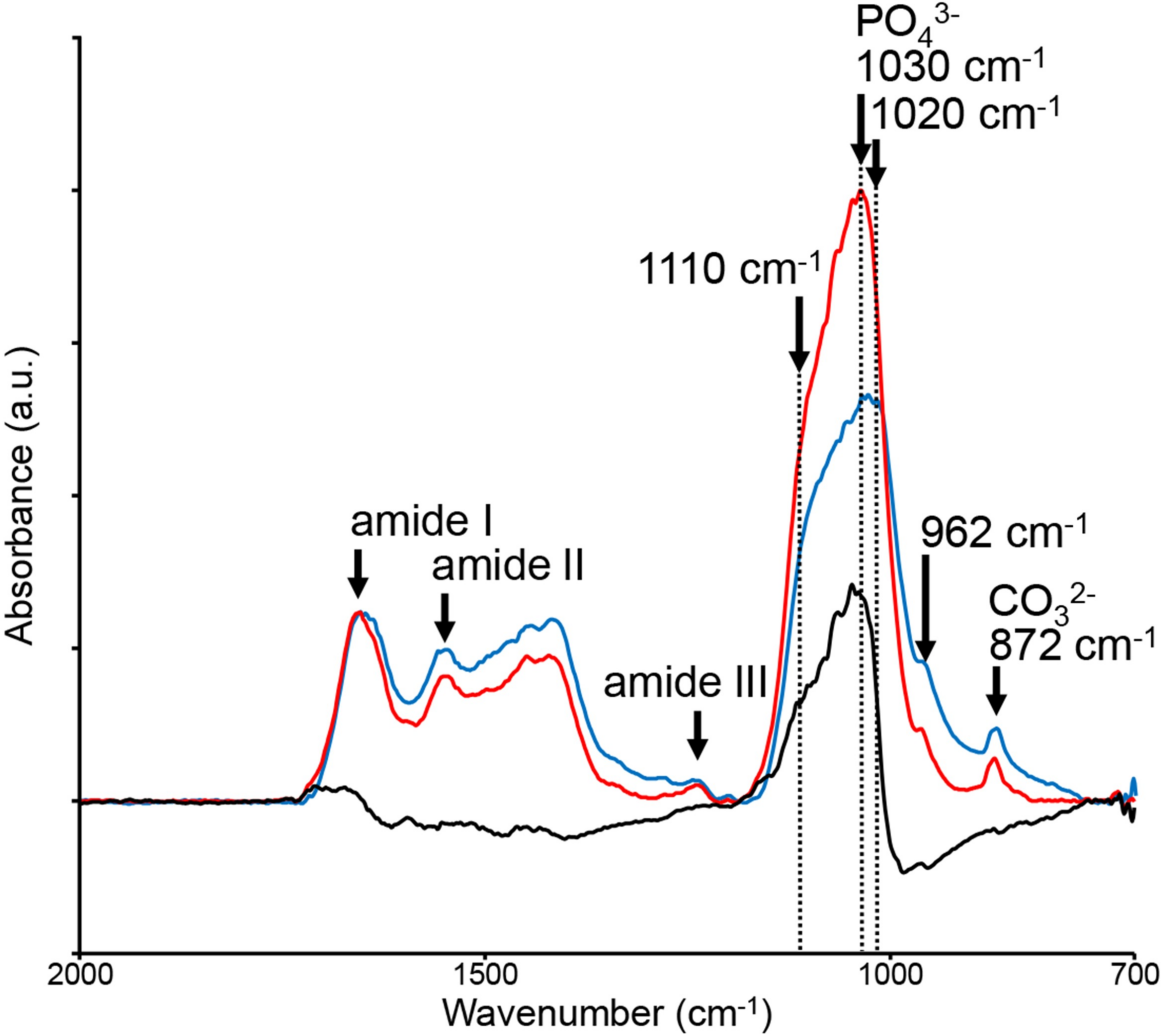
FTIR spectra of RESIN-S and FROZEN-S sections. The red line shows the FTIR spectrum of RESIN-S, and the blue line shows that of FROZEN-S. The spectra were normalized against the amide l band. A subtraction (black line) was obtained by subtracting the FTIR spectrum of the FROZEN-S section from that of the RESIN-S section.

Fig 7 shows the differences in the shape of the FTIR spectrum between the RESIN-S (red line) and the FROZEN-S (blue line) sections, with the PO_4_^3-^ band height and bandwidth for the RESIN-S sections being higher narrower, respectively, than those of the FROZEN-S sections. Absorption at 1030 cm^-1^ (stoichiometric hydroxyapatite [24]) in RESIN-S is significantly increased compared to that in FROZEN-S; however, the amide II and CO_3_^2-^ bands were reduced in RESIN-S.

## Discussion

Bone strength, which is influenced by both BMD and bone quality, is used as an indicator of bone health. Therefore, it is important to measure bone strength in order to estimate the risk of bone fracture. Methods already exist for the clinical measurement of BMD, but we still do not have a useful method for the clinical evaluation of bone quality. Many parameters are known to contribute to bone quality and, therefore, it is necessary to determine several parameters simultaneously, quickly, and easily. FTIR imaging is a powerful tool for the simultaneous characterization of several parameters related to bone quality. On the other hand, FTIR imaging in transmission mode requires the preparation of sufficiently thin bone sections of less than 5 μm. The PMMA embedding procedure is a common method for the preparation of thin bone sections; however, it takes more than two weeks to complete the embedding procedure, and denaturation of proteins within the bone, including collagen, is caused by exposure to the fixing solvent and MMA. The secondary structure of collagen is changed by dehydration [4, 25, 26], and the area or height of the amide I band in the bone after exposure EtOH is subsequently changed [4, 18]. PMMA penetrates into lowly mineralized tissues, and the PMMA spectrum overlap with the mineral and matrix spectra, making subtraction of the spectra more difficult [15]. In the assessment for the bone quality using FTIR imaging, we have to use a quick and easy sample preparation method that reduces the risk of collagen denaturation. We, therefore, used a combination of a PP film + glue substrate and a frozen sectioning procedure. In the FTIR spectrum of the PP film + glue, the most intense absorption bands at 1736 cm^-1^ and 3051–2752 cm^-1^ (CH_2_, CH_3_) were not saturated, indicating that the PP film + glue was thin enough for the IR beam to pass through. Neither the intense (3051–2752 cm^-1^) nor medium bands (1458, 1376, 1255, 1167 cm^-1^) overlapped with the amide I or PO_4_^3-^ bands, while the bands at 997 and 972 cm^-1^ were weak. After subtraction of the PP film + glue from the raw data, the influence of the anisotropy of the PP film was found to be negligible as the absorbance of PO_4_^3-^ was much stronger than that of PP film. Therefore, the direction of the bone mounted on the PP film is considered not to be important, and we can use PP film + glue as a substrate of FTIR imaging.

The bone quality of the femoral thin sections prepared using the two different sectioning procedures (RESIN-S, FROZEN-S) was evaluated by FTIR imaging. Both the absorbance and shape of the PO_4_^3-^ bands differed between the two types of sections. The mineral-to-matrix ratio and crystallinity in the RESIN-S sections were higher than those in the FROZEN-S sections, whereas the carbonate-to-phosphate ratio in the RESIN-S sections was lower than that in the FROZEN-S sections. These results indicated the embedding procedure should be considered when comparing bone quality. The amide I band is overlapped by the water band in both the FTIR and Raman spectra [27]. Based on Raman spectroscopy, Yeni et al. demonstrated that dehydration results in a reduction in the amide I band [4]. The increase in the mineral-to-matrix ratio in the RESIN-S could be caused by a reduction in the amide I band due to dehydration. Crystallinity is changed by the lattice structure, crystal size, and strain environment of the lattice [3, 4]. The pH of MMA is relatively acidic; therefore, the increase in crystallinity and decrease in carbonation during RESIN-S preparation might be consequence of dissolution of poorly crystallized apatite and carbonated apatite [4]. The changes in bone quality obtained from the FTIR spectra corresponded with the results obtained from the Raman spectra by Yeni et al. [4]. Dehydration occurs due to the repeated changes of 100% EtOH during the PMMA embedding procedure [15]. The dehydration during the PMMA embedding procedure might, therefore, cause an enhancement in molecular orientation, thus increasing the dipole moment in hydroxyapatite.

McElderry et al. determined the effects of repeated freeze-thaw on bone quality obtained by Raman measurements, and demonstrated that only the crystallinity value was diminished after one freeze-thaw cycle, with the carbonate-to-phosphate ratio and amide I band not significantly changed [16]. We used fresh bone for the frozen sectioning; that is, the thin sections after one freeze-thaw were determined by FTIR imaging. Consequently, we only need to consider crystallinity when using our method. We previously demonstrated that Raman spectra are more sensitive to crystallinity than FTIR spectra [28]. Therefore, the effects of our frozen process on the assessment of bone quality using FTIR imaging appear to be negligible.

The FTIR image of the RESIN-S sections was clearer than that of the FROZEN-S sections, indicating that the PMMA embedding procedure was suitable for the evaluation of trabecular bone.

The PMMA is better for physical support during the sectioning rather than SCEM for the frozen sectioning. However, the frozen sectioning procedure can reduce the time, cost, and risk of denaturation of proteins by solvent and resin and achieve more accurate measurements compared to the conventional sectioning procedure using PMMA embedding. The PP film is also low cost and has better heat shock resistance, so that the PP film + glue appears to be suitable for use as a substrate for FTIR imaging. Thus, the combined use of PP film + glue and the frozen sectioning procedure affords a powerful technique for FTIR imaging in a clinical setting due to reductions in time and cost. This is the first study on the assessment of the bone quality of fresh calcified bone using FTIR imaging in transmission mode. Further work is needed to determine the stability of fresh bone stored in a freezer in order to make this technique more useful.

## Conclusions

In conclusion, we demonstrated that the combined use of 4-μm-thick PP film coated with glue as a substrate and a frozen sectioning procedure without denaturation of proteins by solvent and resin was well suited to the assessment of the bone quality of fresh calcified bone using FTIR imaging in transmission mode, and the newly developed sample preparation procedure affords a powerful tool for clinical evaluation due to reductions in time and cost. The conventional PMMA embedding procedure for bone takes more than two weeks and exposes the bone to high concentration of EtOH and MMA, which cause denaturation of proteins including collagen; however, the PMMA is better for physical support, and the FTIR image of trabecular bone in the sections embedded in PMMA was clearer than that in the frozen sections. Therefore, the combined use of the coated PP film and the frozen sectioning procedure affords a more accurate method for the evaluation of true bone quality.

This is the first study on the assessment of the bone quality of fresh calcified bone using FTIR imaging in transmission mode. Further work is needed to determine the stability of fresh bone stored in a freezer in order to make this technique more useful.

## Supporting information

S1 Fig FTIR spectra of the femur section before and after rinsing with water. The blue line shows the FTIR spectrum of the femur section on the PP film + glue before rinsing with water, and the red line shows that after rinsing. The spectra were normalized against the PO_4_^3-^ band. There was no significant difference in the shape of FTIR spectra before and after rinsing with water.
(TIF)

S2 Fig A representative FTIR spectrum of the femur section on the PP film + glue. The FTIR spectrum of the femur section on the PP film was not saturated in the region from 2000 cm^-1^ to 700 cm^-1^.
(TIF)

## References

[1] Burr DB, Akkus O. Bone Morphology and Organization In: Burr DB, Allen MR, editors. Basic and Applied Bone Biology. 1st ed. USA: Academic Press; 2013 p. 3–25.

[2] Wopenka B, Pasteris JD. A mineralogical perspective on the apatite in bone. Materials Science and Engineering: C. 2005;25(2):131–43. doi: 10.1016/j.msec.2005.01.008

[3] Farlay D, Panczer G, Rey C, Delmas PD, Boivin G. Mineral maturity and crystallinity index are distinct characteristics of bone mineral. Journal of bone and mineral metabolism. 2010;28(4):433–45. doi: 10.1007/s00774-009-0146-7 20091325 10.1007/s00774-009-0146-7 PMC2958843

[4] Yeni YN, Yerramshetty J, Akkus O, Pechey C, Les CM. Effect of fixation and embedding on Raman spectroscopic analysis of bone tissue. Calcified tissue international. 2006;78(6):363–71. doi: 10.1007/s00223-005-0301-7 16830201 10.1007/s00223-005-0301-7

[5] Kimura-Suda H, Kuwahara M, Hidaka K, Kanazawa K, Honma K, Ueno H, et al Analysis of Bone in Rats of End Stage Kidney Disease by Vibrational Spectroscopy. Mol Cryst Liq Cryst. 2012;566(1):75–9. doi: 10.1080/15421406.2012.701831

[6] Kimura-Suda H, Kajiwara M, Sakamoto N, Kobayashi S, Ijiro K, Yurimoto H, et al Studies on bone metabolism by using isotope microscopy, FTIR imaging, and micro-Raman spectroscopy. J Oral Biosci. 2013;55(2):61–5. doi: 10.1016/j.job.2013.04.006

[7] Kimura-Suda H, Ito T, Kanazawa K, Chaki N, Akiyama H. Collagen Fiber Orientation in the Femur of Rats with Chronic Kidney Disease. e-JSSNT. 2015;13(0):244–6. doi: 10.1380/ejssnt.2015.244

[8] Ito T, Kanazawa K, Kimura-Suda H. Analysis of Collagen Fiber Orientation in Bone of Different Aged Rats Using FTIR Imaging. Mol Cryst Liq Cryst. 2015;622(1):114–9. doi: 10.1080/15421406.2015.1097011

[9] Morris MD, Schulmerich MV, Dooley KA, Esmonde-White KA. Vibrational Spectroscopic Imaging of Hard Tissues In: Salzer R, Siesler HW, editors. Infrared and Raman Spectroscopic Imaging. 1st ed. Weinheim: Wiley-VCH; 2009 p. 149–71.

[10] Rehman IU, Movasaghi Z, Rehman S. Chemical Structural Analysis of Bone by Spectroscopy. USA: Taylor & Francis; 2012 189–211 p.

[11] Farlay D, Duclos ME, Gineyts E, Bertholon C, Viguet-Carrin S, Nallala J, et al The ratio 1660/1690 cm(-1) measured by infrared microspectroscopy is not specific of enzymatic collagen cross-links in bone tissue. PloS one. 2011;6(12):e28736 doi: 10.1371/journal.pone.0028736 22194900 10.1371/journal.pone.0028736 PMC3237494

[12] Acerbo AS, Carr GL, Judex S, Miller LM. Imaging the material properties of bone specimens using reflection-based infrared microspectroscopy. Analytical chemistry. 2012;84(8):3607–13. doi: 10.1021/ac203375d 22455306 10.1021/ac203375d PMC3364542

[13] Nicholson CL, Firth EC, Waterland MR, Jones G, Ganesh S, Stewart RB. Innovative Approach to Investigating the Microstructure of Calcified Tissues Using Specular Reflectance Fourier Transform-Infrared Microspectroscopy and Discriminant Analysis. Analytical chemistry. 2012;84(7):3369–75. doi: 10.1021/ac300123r 22413951 10.1021/ac300123r

[14] Gadeleta SJ, Boskey AL, Paschalis E, Carlson C, Menschik F, Baldini T, et al A physical, chemical, and mechanical study of lumbar vertebrae from normal, ovariectomized, and nandrolone decanoate-treated cynomolgus monkeys (Macaca fascicularis). Bone. 2000;27(4):541–50. doi: 10.1016/S8756-3282(00)00362-8 11033450 10.1016/s8756-3282(00)00362-8

[15] Aparicio S, Doty SB, Camacho NP, Paschalis EP, Spevak L, Mendelsohn R, et al Optimal methods for processing mineralized tissues for Fourier transform infrared microspectroscopy. Calcified tissue international. 2002;70(5):422–9. doi: 10.1007/s00223-001-1016-z 12055658 10.1007/s00223-001-1016-z

[16] McElderry JD, Kole MR, Morris MD. Repeated freeze-thawing of bone tissue affects Raman bone quality measurements. Journal of biomedical optics. 2011;16(7):071407 doi: 10.1117/1.3574525 21806253 10.1117/1.3574525 PMC3144971

[17] Boskey AL, Cohen ML, Bullough PG. Hard tissue biochemistry: a comparison of fresh-frozen and formalin-fixed tissue samples. Calcified tissue international. 1982;34(4):328–31. doi: 10.1007/BF02411262 6814720 10.1007/BF02411262

[18] Pleshko N, Boskey A, Mendelsohn R. An FT-IR microscopic investigation of the effects of tissue preservation on bone. Calcified tissue international. 1992;51(1):72–7. doi: 10.1007/BF00296221 1393781 10.1007/BF00296221

[19] Pleshko NL, Boskey AL, Mendelsohn R. An infrared study of the interaction of polymethyl methacrylate with the protein and mineral components of bone. The journal of histochemistry and cytochemistry: official journal of the Histochemistry Society. 1992;40(9):1413–7. doi: 10.1177/40.9.1506677 1506677 10.1177/40.9.1506677

[20] Boucek RJ, Noble NL, Gunja-Smith Z. A possible role for dehydrodihydroxylysinonorleucine in collagen fibre and bundle formation. The Biochemical journal. 1979;177(3):853–60. 444208 10.1042/bj1770853 PMC1186450

[21] Hanifi A, McGoverin C, Ou YT, Safadi F, Spencer RG, Pleshko N. Differences in infrared spectroscopic data of connective tissues in transflectance and transmittance modes. Analytica chimica acta. 2013;779:41–9. doi: 10.1016/j.aca.2013.03.053 23663670 10.1016/j.aca.2013.03.053 PMC3900307

[22] Bairi VG, Lim JH, Quevedo IR, Mudalige TK, Linder SW. Portable X-ray fluorescence spectroscopy as a rapid screening technique for analysis of TiO2 and ZnO in sunscreens. Spectrochim Acta Part B At Spectrosc. 2016;116:21–7. doi: 10.1016/j.sab.2015.11.008 27076699 10.1016/j.sab.2015.11.008 PMC4827927

[23] Iu Rehman, Movasaghi Z Rehman S. FTIR and Raman Characteristic Peak Frequencies in Biological Studies Chapter 8 FTIR and Raman Characteristic Peak Frequencies in Biological Studies: CRC Press; 2012 p. 213–94.

[24] Rey C, Shimizu M, Collins B, Glimcher MJ. Resolution-enhanced fourier transform infrared spectroscopy study of the environment of phosphate ion in the early deposits of a solid phase of calcium phosphate in bone and enamel and their evolution with age: 2. Investigations in the v3 PO4 domain. Calcified tissue international. 1991;49(6):383–8. doi: 10.1007/BF02555847 1818762 10.1007/BF02555847

[25] Lees S. A mixed packing model for bone collagen. Calcified tissue international. 1981;33(6):591–602. Epub 1981/01/01. doi: 10.1007/BF02409497 6799171 10.1007/BF02409497

[26] Lees S, Heeley JD, Cleary PF. Some properties of the organic matrix of a bovine cortical bone sample in various media. Calcified tissue international. 1981;33(1):83–6. doi: 10.1007/BF02409417 6780159 10.1007/BF02409417

[27] Miller LM, Bourassa MW, Smith RJ. FTIR spectroscopic imaging of protein aggregation in living cells. Biochimica et biophysica acta. 2013;1828(10):2339–46. doi: 10.1016/j.bbamem.2013.01.014 23357359 10.1016/j.bbamem.2013.01.014 PMC3722250

[28] Kimura-Suda H, Ito T. Bone quality characteristics obtained by Fourier transform infrared and Raman spectroscopic imaging. J Oral Biosci. 2017;59:142–5. doi: 10.1016/j.job.2017.04.002

