# Supplementary figures and images for: Quick and easy sample preparation without resin embedding for the bone quality assessment of fresh calcified bone using fourier transform infrared imaging
Source: PLoS One. 2018 Feb 6;13(2):e0189650. doi: 10.1371/journal.pone.0189650 (PMC5800566; doi:10.1371/journal.pone.0189650)

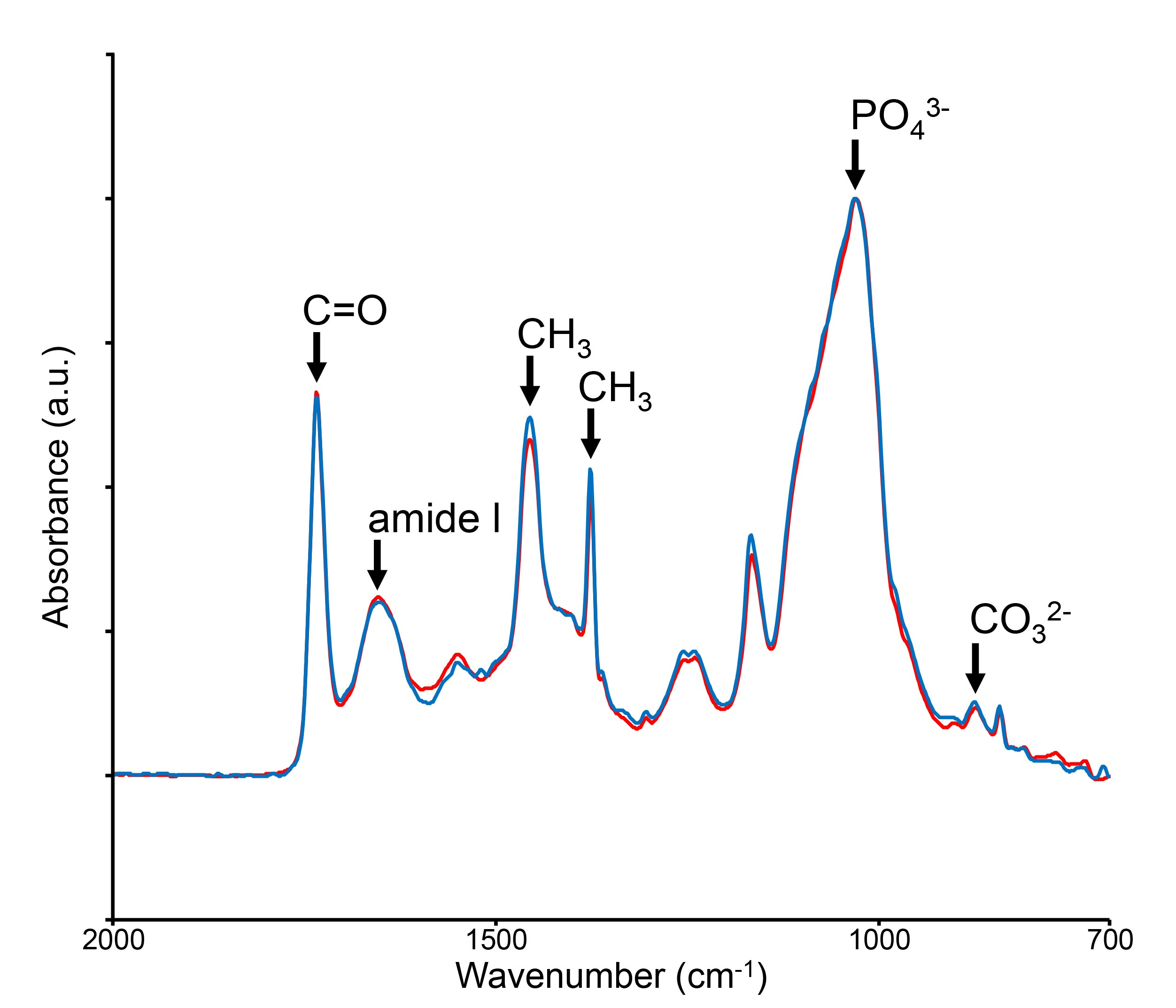

Supplement: S1 Fig — The blue line shows the FTIR spectrum of the femur section on the PP film + glue before rinsing with water, and the red line shows that after rinsing. The spectra were normalized against the PO43- band. There was no significant difference in the shape of FTIR spectra before and after rinsing with water. (TIF) [file pone.0189650.s001.tif]

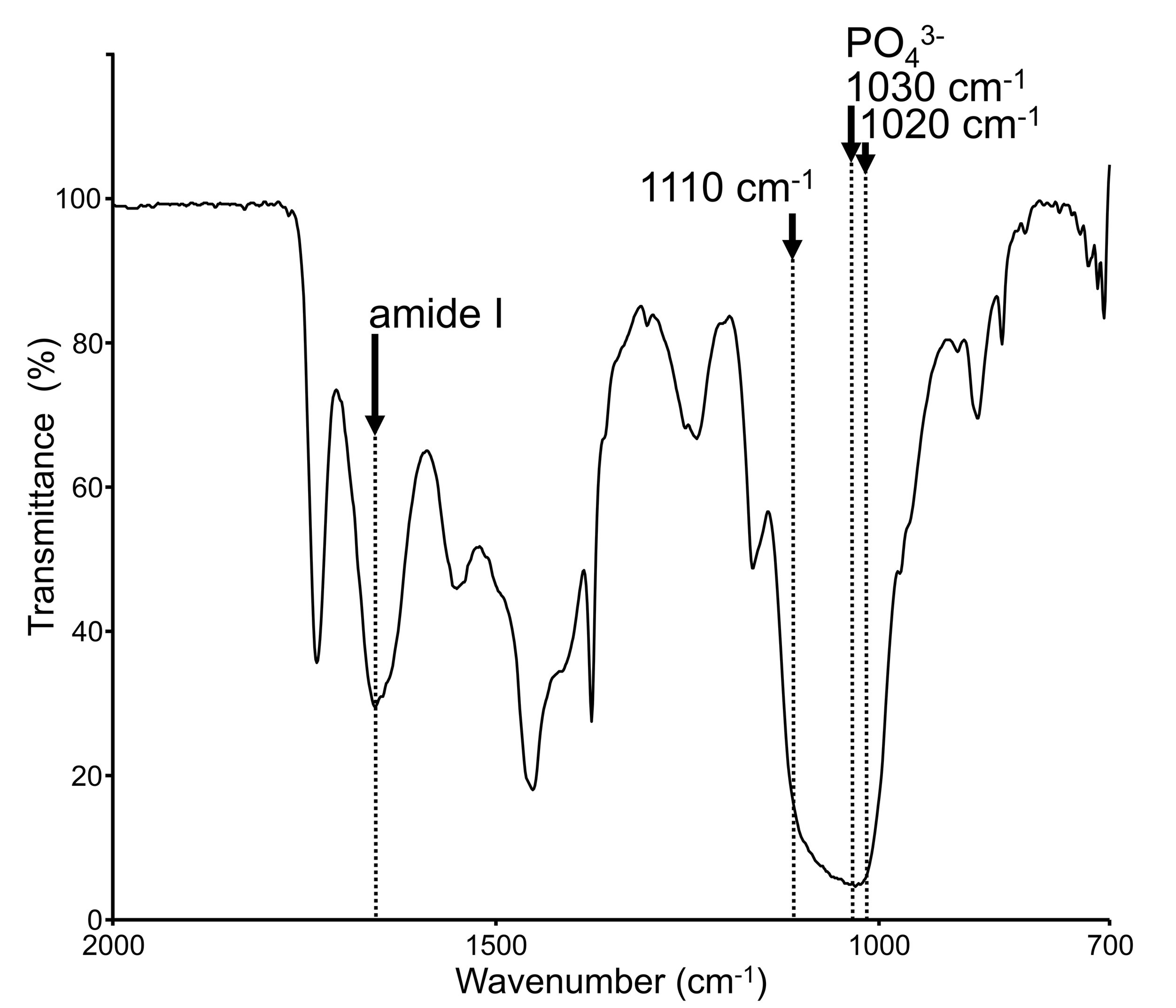

Supplement: S2 Fig — The FTIR spectrum of the femur section on the PP film was not saturated in the region from 2000 cm-1 to 700 cm-1. (TIF) [file pone.0189650.s002.tif]
